# Current understanding of macrophages in intracranial aneurysm: relevant etiological manifestations, signaling modulation and therapeutic strategies

**DOI:** 10.3389/fimmu.2023.1320098

**Published:** 2024-01-08

**Authors:** Jian Duan, Qijie Zhao, Zeyuan He, Shuang Tang, Jia Duan, Wenli Xing

**Affiliations:** ^1^ Department of Cerebrovascular Disease, Suining Central Hospital, Suining, Sichuan, China; ^2^ Department of Pharmacy, West China Hospital, Sichuan University, Chengdu, Sichuan, China

**Keywords:** intracranial aneurysm (IA), macrophages, inflammation, cellular signaling, therapeutic strategy

## Abstract

Macrophages activation and inflammatory response play crucial roles in intracranial aneurysm (IA) formation and progression. The outcome of ruptured IA is considerably poor, and the mechanisms that trigger IA progression and rupture remain to be clarified, thereby developing effective therapy to prevent subarachnoid hemorrhage (SAH) become difficult. Recently, climbing evidences have been expanding our understanding of the macrophages relevant IA pathogenesis, such as immune cells population, inflammatory activation, intra-/inter-cellular signaling transductions and drug administration responses. Crosstalk between macrophages disorder, inflammation and cellular signaling transduction aggravates the devastating consequences of IA. Illustrating the pros and cons mechanisms of macrophages in IA progression are expected to achieve more efficient treatment interventions. In this review, we summarized the current advanced knowledge of macrophages activation, infiltration, polarization and inflammatory responses in IA occurrence and development, as well as the most relevant NF-κB, signal transducer and activator of transcription 1 (STAT1) and Toll-Like Receptor 4 (TLR4) regulatory signaling modulation. The understanding of macrophages regulatory mechanisms is important for IA patients’ clinical outcomes. Gaining insight into the macrophages regulation potentially contributes to more precise IA interventions and will also greatly facilitate the development of novel medical therapy.

## Introduction

The global prevalence of current intracranial aneurysm (IA) is approximate 3.2% (1). Saccular IA is a pathological dilation at major branching brain arteries (2). Generally, more than 80% of saccular IA is acquired lesions within the anterior circulation, and is characterized by an out-bulging within the thinning arterial wall region. Recent high-resolution magnetic resonance imaging (HR-MRI) technology shows a great potential in detecting unstable and unruptured IA (3). The intracranial aneurysm rupture will cause subarachnoid hemorrhage (SAH) and classic presentation of a thunderclap headache, which has a poor outcome and presented with a severe mortality more than 25% (4). According to the current understanding, IA is not a congenital disorder, but develops over the life course, where hemodynamic stress, vascular risk (hypertension, lipid accumulation, arteriosclerosis and smoking), genetic and environmental risk factors are involved in the process of IA (5). In a prospective study with 30 IA patients, enhanced-MRI visually shows macrophages as markers of inflammation in the aneurysm wall (6). Degeneration or disruption of the internal elastic lamina at an arterial bifurcation is a key event in IA out-bulging formation (7). The IA growth is discontinuous and stochastic rather than linear, which remains unchanged and inflammatory microenvironment for a long time before undergoing episodes of rapid growth and easy rupturing (8). Some IA seems to have no symptom when measured at a size of 7 mm or less (1), and natural pathological mechanisms of IA remain poorly understood. Therefore, it is still necessary to understand the pathophysiological processes and develop treatment strategies for IA.

IA is associated with complex pathological changes characterized by hemodynamics, genetics, inflammatory and immune response in cerebral arteries (9). Of note, the high wall shear stress in IA site has been reported to evoke endothelial cells inflammatory response and immune cells accumulation, subsequently spreading to more arterial wall region under the stimulation of macrophages and proinflammatory factors secretion (10). Due to macrophages are prevalently observed in human IA, they have gained wide attention in the IA pathogenesis. Recent studies have shown that macrophages infiltration and relevant cellular and molecular regulations are important in IA progression (11). The macrophages-induced cytokines and matrix metalloproteinases (MMPs) showed the ability to digest extracellular matrix and thin the vessel wall, consequently promoting the aneurysmal formation (12). There are mounting clues indicated that inflammatory macrophages are the dominant factor in IA, while macrophages microenvironment is complex and the pathogenic mechanisms are still poorly understood. Of note, various evidences indicated a causal link between the cellular signaling pathway and IA progression, such as C-C motif chemokine ligands (CCLs), NF-κB, signal transducer and activator of transcription 1 (STAT1), and toll like receptor 4 (TLR4) signaling (13–15). Although the current diagnosis and treatment technology have made significant progress, further research is needed to improve the inflammatory response and progression of IA with specific signaling target interventions. A safe and noninvasive therapy strategy is urgently needed to prevent IA progression and rupture. To achieve this goal, the underlying mechanisms by which macrophages cause the initialization and development of IA should be elucidated.

In this review, we summarized the macrophages abnormalities and inflammation regulations during the occurrence and progression of IA. According to recent thought-provoking studies, etiology and molecular basis of macrophages relevant IA are highlighted through different prospective of intracellular and extracellular regulation in this review. Several pivotal manifestations in macrophages activation, polarization, lipids metabolism and signaling transduction (NF-κB, STAT1, and TLR4) were also discussed in our study. Understanding the macrophages relevant mechanisms in specific IA pathogenesis is important for developing therapeutic strategies to prevent disease development and brain injury.

## Macrophages recruitment and activation in IA

**Figure 1:**
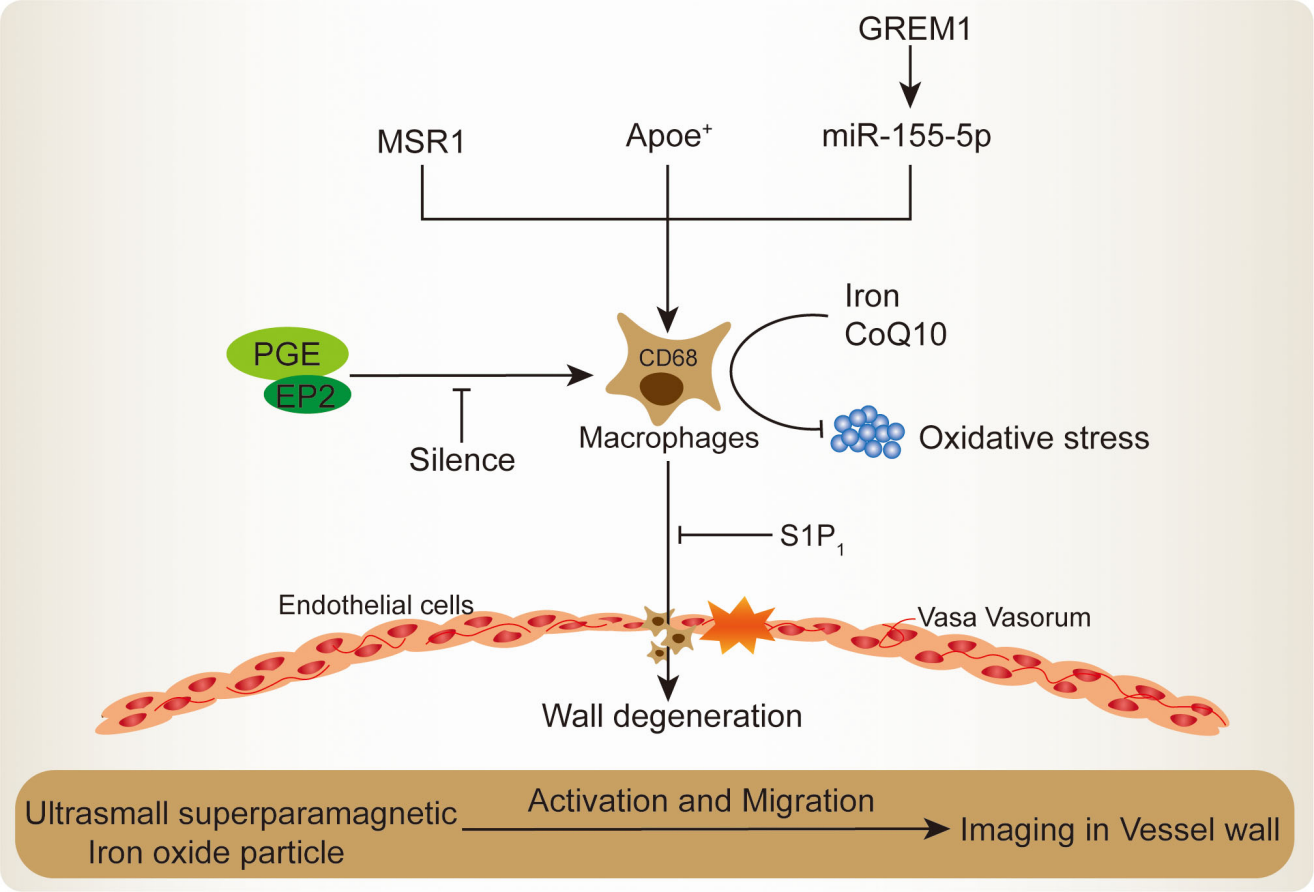
Mechanisms of macrophages recruitment and activation in IA lesion.

Sever morphological changes and significantly increased macrophages were observed in IA, wherein the increased M2/M1 ratio seems to be the hallmark of ruptured IA (16). Noteworthily, higher proportion of M2 macrophages was observed in ruptured IA compared with unruptured IA. In both animal models and human IA (**Figure 1**), high recruitment and infiltration of macrophages have been observed in aneurysm walls (17). On the other hand, the exhaustion of macrophages showed the ability to reduce the IA incidence (18). Due to the intracranial arteries lack vasa vasorum in the adventitia, macrophages and other immune inflammatory cells could pass through the endothelial cells and infiltrate into aneurysm walls (18, 19). Of note, increased macrophages marker CD68 expression has been observed in cerebral arteries smooth muscle cells and along with CXCL1, monocyte chemoattractant protein 1 (MCP-1), TNF-α upregulation (20). Different subtypes of macrophages presented with asymmetrical distribution, where CD68^+^ macrophages have trended towards predominance within myointimal hyperplasia region rather than CD163^-^ macrophages (21). The higher CD68^+^ macrophages accumulation in IA region was accompanied with higher density of mast cells and neo-vessels, as well as being associated with disease progression (22). Recent single-cell transcriptome evidence demonstrated that ApoE^+^ macrophages presented with overwhelming infiltration in some IA, and macrophage-derived ApoE potentially served as a biomarker to distinguish IA in molecular pathology (23). In vascular diseases, ApoE has been reported to reduced smooth muscle cells proliferation and be involved in phenotypic remodeling (24). As a lipid transport molecule, the ApoE relevant lipid efflux is a feature of peripheral vascular structural variations and aneurysm lesions (25, 26). Moreover, with various macrophage phenotypes gene signatures, single-cell transcriptome evidences profiled more macrophage subtypes like macrophage-type5 (HLA-DQAs/MRC1) and macrophage-type6 (C1QA/C1QB/CD74) (23). These two types macrophages were presented with chemotaxis and exhibited antigen-presenting effects, particularly involved in IA progression and inflammation. Moreover, the mouse model based single cell transcriptome evidences indicated that IA induction led to significant expansion of the total macrophage populations, as well as macrophages further expansion after rupture (27). Among which, six macrophage subtypes were identified in IA and act as major source of vascular inflammation. ApoE/Wfdc17/Pf4 enriched macrophages were proved to promote cytokine, inflammatory responses and differentiation, Ly6c2/Chil3/Plac8 enriched macrophage presented with higher infiltrating ability, MHC-II/cd74positive macrophages were more likely contribute to IA-associated acute injury. On the other hand, high levels of fatty acid β-oxidation and OXPHOS macrophages in IA were categorized in M2-like macrophages, which were related to ribosomal/mitochondrial activation and had lower levels of inflammatory stimulus (27). During the IA progression and rupture, dramatic change in the macrophages is complex in vascular wall lesion region and inflammation, the discrepancy is likely due to the macrophage heterogeneity and requires further investigation (27, 28).

Macrophage-derived exosomal miR-155-5p, an antagonist target for bone morphogenetic protein Gremlin 1 (GREM1) secretion, which shows the ability to stimulate macrophages activation and infiltration by promoting smooth muscle cells migration in IA (29). The macrophages migration and activation in IA will further impair the endothelial cells inter-cellular junction, consequently leading to the severe structural lesions (17). Higher macrophages accumulation will lead to degenerative wall remodeling in IA and make it predisposing to rupture (21). In terms of this, the sphigosine-1-phosphate receptor type 1 (S1P_1_) potentially strengthens the endothelial cells structural integrity and simultaneously reduces macrophages infiltrating, whose activation will regress the IA out-bulging lesion (30). The S1P_1_ has been reported to inhibit vascular inflammation and lesion, which regulates vascular development and microvascular barrier function (31). S1P_1_ agonist shows a promising future for the treatment of ischemic and hemorrhagic stroke (32). Similarly, prostaglandin E (PGE) receptor subtype 2 (EP_2_), its specifically silence could almost completely suppress macrophages infiltration and inflammatory responses in IA (10). The EP2 comprises G protein–coupled receptors to recognize PGE and plays an important role in the modulation of blood pressure and inflammatory response (33). The PGE implicated in the vascular remodeling in response to inflammation and hypertension (34). Injured vascular region presented with PGE secretion and modulated vascular smooth muscle cell proliferation, and PGE-induced growth state-dependent actions was mediated via the receptor EP_2_ (35). Inhibition of EP_2_ could decrease PGE activation and inflammation microvascular dysfunction in the brain retinopathy (36). In contrast, within the IA, the EP_2_-induced macrophages self-amplification loop will promote macrophages activation and infiltration in the out-bulging lesions (10). Recently, transcriptome and functional experimental evidences suggested that macrophages are abnormally enriched in IA lesions, wherein the core macrophage scavenger receptor 1 (MSR1) gene was demonstrated to be associated with its activation and migration (9). The MSR1 named CD204, was primarily found on the various types of macrophage surface and was responsible for the pro-inflammatory polarization both *in vivo* and *in vitro* (37, 38). In the aspect of nutrition improvement, recent studies demonstrated that iron limitation and coenzyme Q10 (CoQ10) administration significantly ameliorated the macrophages infiltration and oxidative stress, thereby attenuating IA progression and rupture (39, 40). Through engulfed ultrasmall superparamagnetic iron oxide particle, the macrophages enrichment vessel wall imaging in IA has potential in qualitative evaluation and can be used to evaluate the efficacy of medication (41).

In the clinical practice, thickening of the vessel wall and IA were accompanied with abundant infiltration of inflammatory cells like macrophages. The macrophages infiltrating and inflammation around the sites of neovascularization play an important role in the pathophysiology of IA, especially promoting aneurysm wall degeneration (20). Macrophages recruitment and activation could imply degeneration of the aneurysm wall in remodeling process with inflammation. However, the IA progression and rupture could be various and have been explained on the unstable vascular tissue environment and hemodynamics (5). According to the current histopathologic studies, aneurysm walls are individually heterogeneous, and individualized treatment options remain to be addressed. The submillimeter structures of small IA were inadequate to visualize all the components under the limited spatial resolution (42). The proteases and other matrix degrading enzymes secreted by macrophages would demolish extracellular matrix and destabilize the IA wall (43, 44). Due to the differential characteristics of macrophages functions in IA and limited clinical validation size, complex regulation is exhibited by complex stimuli of multiple factors rather than single factor stimuli, making clinical translation difficult (1). Some IA may rupture regardless of the size in the early phase or enlarge in a short time because of excessive wall thinning by advancement of degenerative changes, but currently no accurate methods are available for predicting which patients are at macrophages-induced IA rupture risk and benefit from surgical or endovascular intervention (45). On the other hand, many reports have been conducted only on macrophages, while many types of immune cells interaction and working together in the IA symptomatic responses still need further investigation, such as T lymphocytes and mast cells (27, 46, 47).

Macrophages contribute not only to the pathogenesis of IA but also various other important vascular diseases. In the abdominal aortic aneurysms (AAA), CD11c positive macrophages were present throughout the diseased region (48). With the Th1 immune responses, the cytokines induced macrophages would generate central proteases MMP-3 and MMP-9 to remodel AAA vascular wall matrix (49). In terms of this, the TRAP-positive macrophages were demonstrated to increase MMP-9 expression and were positively associated with AAA progression (50, 51). The AAA local inflammatory environment undergoes macrophages accumulation and polarization, as well as cytokines TNF-α, IL6, IL-12 and IL-1β secretion (52, 53). Different from IA, the aortic walls undergo a switch from M1 macrophage phenotype to M2 macrophage phenotype during AAA progression, and presented a compensatory mechanism of the anti-inflammatory and tissue-repair effect (54). Moreover, with the JNK and p38 pathways regulation, macrophage-derived exosomes could stimulate MMP-2 in vascular smooth muscle cells and contribute to the AAA development (53). Noteworthy, Previous mouse aneurysm model study indicated that higher macrophages migration was presented in AAA when compared with thoracic aortas aneurysms (TAA) (55). Similarly, in both AAA and TAA, the CCN4 blocking significantly reduced the macrophages migration and activation rather than smooth muscle cells, thereby decreasing the number of ruptured aortae (56). In the TAAs with dilatation ≤ 6cm, the upregulated CD68^+^ macrophages accumulation was upregulated in the lesion region and mainly accompanied with huge amounts of MMP-9 production, while MMP-2 only has a slight elevation (57). Through disrupting the extra-cellular matrix components and aortic wall, elevated levels of extracellular metalloproteinases and macrophages will further facilitate TAA inflammation and progression (58). On the contrary, with the inhibition of NLRP3/IL-1β inflammatory signaling, macrophages and MMP-9 levels could be restricted in TAA (59, 60). The decreasing of macrophages and extracellular metalloproteinases significantly attenuated TAA formation and progression (61). Moreover, in the β-aminopropionitrile fumarate (BAPN) induced TAA, macrophages were predominantly in the G0/G1 phase and potentially be inhibited by SIRT1 signaling activation (62). Although both AAA and TAA are characterized by progressive dilation of the aortic wall, detail molecular mechanisms underlying TAA have some different and more likely develop structural instability (63). The familial TAA is predominantly caused by genomic alteration that encodes extracellular matrix proteins and stimulates TGF-β signaling pathway, while the sporadic TAA presented with increased inflammation and vascular degradation (63, 64).In addition, another fatal vessel vascular disease aortic dissection (AD) also presented with inflammation and structural destroy, wherein macrophages are the hub of inflammation in the aortic wall and angiotensin II (Ang II) has been shown to be an important factor for macrophages stimulation (65, 66). Macrophages infiltration may be more severe in AD than in aortic aneurysm and is critical for early AD formation (67). In the Ang II induced macrophages accumulation, the FKBP11/NF-κB cascade and SMAD4 mutation were involved in macrophages infiltration and M1 differentiation, thereby further promoting MMPs secretion (68, 69). In addition, upstream Th-17/IL-17 axis is another regulator for Ang II-induced macrophages inflammation and aortic wall remodeling (70). In most recent studies, NLRP3, Nrg4, JAK2, CD31 disregulation were emerged to be positively associated with macrophages activation and AD inflammation (71–74). Recovering the pro- and anti-inflammatory macrophages balance would ameliorate AD lesion. Of note, highly increased granulocyte macrophage colony-stimulating factor (GM-CSF) is a triggering molecule for AD progression and may be important for diagnostic and therapeutic exploitation (75, 76). On the other hand, the macrophage metabolic reprogramming will activate HIF-1α/ADAM17 signaling and promote AD inflammation and progression (77) Taken together, the macrophages share the similar manifestation in vascular aberration disease, but the underlying mechanisms still need to be further identified in different perspectives.

## Macrophages relevant inflammatory response in IA

**Figure 2:**
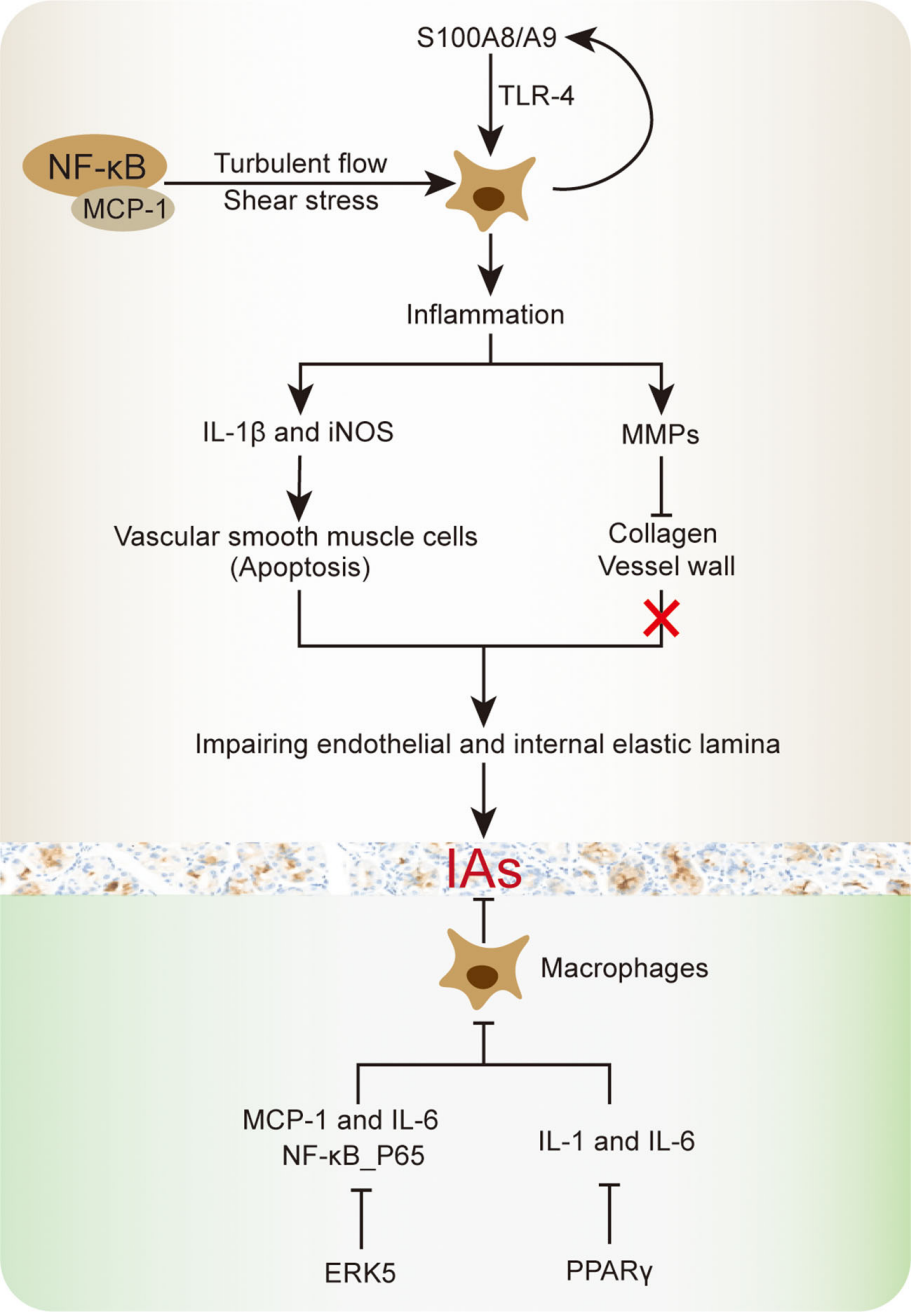
Schematic summary of macrophage inflammation involved in IA progression.

Finding from previous studies suggested that macrophages mediated cellular and molecular inflammation are closely involved in IA progression and rupture, including cytokine and proteinase production (**Figure 2**). Chronic inflammation due to macrophages in vascular wall is a fundamental mechanism in the enlargement of IA (78). Transcriptomic analysis of IA revealed that upregulation of pro-inflammatory cytokine genes was associated with macrophages. For instance, the activation of NF-κB and MCP-1 were involved in macrophages-derived IA inflammation, as well as regulating inflammation associated genes, such as interleukin (IL)−1β, inducible nitric oxide synthase (iNOS) and MMPs (79). Among which, the NF-κB is responsible for MCP-1 upregulation, which cooperated with turbulent flow low wall shear stress to exacerbate macrophages inflammation (80, 81). The increased IL-1β and iNOS have been reported to cause vascular smooth muscle cells apoptosis, thereby impairing endothelial and internal elastic lamina in IA progression (82). Meanwhile, the MMP family member MMP-2 and MMP-9 were supposed to participate in collagen degradation and breakdown relevant vessel wall remodeling, while the MMP-12 did not involve in IA macrophages inflammatory response (18, 44). In this aspect, as an up-stream regulator, EP_2_-induced NF-κB singling activation will further maintain macrophages inflammatory responses in IA lesions (10). However, in compared with other pro-inflammatory cytokines, NF-κB inflammatory manifestation is more inferior than TNF-α. In addition, the peroxisome proliferator-activated receptor-γ (PPARγ)-mediated cellular IL-1 and IL-6 reduction will contribute to pro-inflammatory M1 macrophages regression, which reduced the IA rupture risk in human (81, 83). Even in ruptured IA with severe morphological changes and higher inflammatory cells amount, PPARγ activation could reduce M1/M2 macrophage ratio and improve the inflammatory response (83).

Of note, Taichi et, al. reported that macrophages extracellular signal-regulated kinase 5 (ERK5) activation could inhibit the MCP-1 and IL-6 generation in IA and promote anti-inflammatory effects, thereby shrinking the out-bulging lesions (84). Additionally, ERK5 activation also shows the ability to suppress the phosphorylated NF-κB subunit *in vitro*. The serum NF-κB concentrations were positively correlated with the number and pro-inflammatory response in aneurysms (85–87). Through clinical biospecimen comparison, higher S100A8/A9 protein complex concentration was observed in IA, which will induce macrophages inflammation and vessel wall degeneration (88). Continuous decrease tensile strength in IA wall will accelerate the rupture ending. The S100A8/A9 complex is an adverse cardiovascular events indicator, where proteins S100A8 and S100A9 are recognized to form a heterodimer, they are released by neutrophils, monocytes, and activated macrophages (89, 90). The neutrophilic inflammation can accelerate the macrophages inflammatory process (78). Moreover, the S100A8/A9 is a well-known pro-inflammatory mediator that binds to toll-like receptor 4 (TLR-4) and advanced glycation receptor, consequently promoting various types of cells activation and resulting in reactive oxygen species, cytokines and enzymes upregulation in IA (91, 92). As a cellular surface protein, TLR-4 not merely contributes to innate immune responses, but also promotes macrophages-derived inflammatory processes in IA (13). The TLR-4 induced macrophages inflammatory response will increase the IA rupture rate, as well as enhancing TNF-α, IL-1β, and IL-6 generation. The above mechanism was might stimulated by TLR4 adaptor protein MyD88, which shows a great potential in improving IA progression (13). Taken together, restricting the macrophages-derived inflammation might be a promising approach to prevent IA rupture and subsequent SAH.

## Inducing alternative polarization of macrophages

Monocytes could differentiate into heterogeneous cells. Differential expression of specific membrane molecules underlies artificial division basis for monocyte subsets identification in both human and mouse (93). M1 macrophages were mainly differentiated from Ly6C^high^ monocytes, while the M2 macrophages derived from Ly6C^low^ monocytes (94). Moreover, macrophages were also skewed during differentiation, and the resultant phenotype is delivered on the microenvironment cytokines (95). Among which, the pro-inflammatory TNF-α, TLR4, Myd88, NF-κB were associated with M1 macrophages, whereas PPARγ and ERK5 were involved in M2 macrophages (96). Sufficient evidences indicated that GM-CSF contributed to M1 polarization in IA, which was positively correlated with out-bulging lesions volume, especially IA volume larger than 7 mm (20, 97, 98). The blocking of GM-CSF significantly inhibited M1 macrophages and MMP-9 secretion (75). In addition, the STAT signaling is an important clue in modulating macrophage M1/M2 polarization (99, 100). The intervention of STAT signaling could significantly decrease the M1 macrophages polarization in IA (15). During the IA progression, inhibition of CXCL1 reversed the macrophages M1-like polarization (101). The CXCL1-induced pro-inflammatory neutrophils were observed to promote the macrophage inflammatory protein-1α (MIP-1α/CCL3) secretion, subsequently polarizing macrophages into the M1 phenotypes (102, 103). More recently, the receptor tyrosine kinase Axl was demonstrated to promote M1 macrophages polarization in IA, wherein the STAT1 knockdown showed the ability to abolish above polarization process (11). The activated STAT1/HIF-1α signaling might be responsible for Axl-mediated M1 phenotype polarization paradigm and served as downstream of Axl signaling, ultimately increasing the IA rupture risk (11).

**Figure 3:**
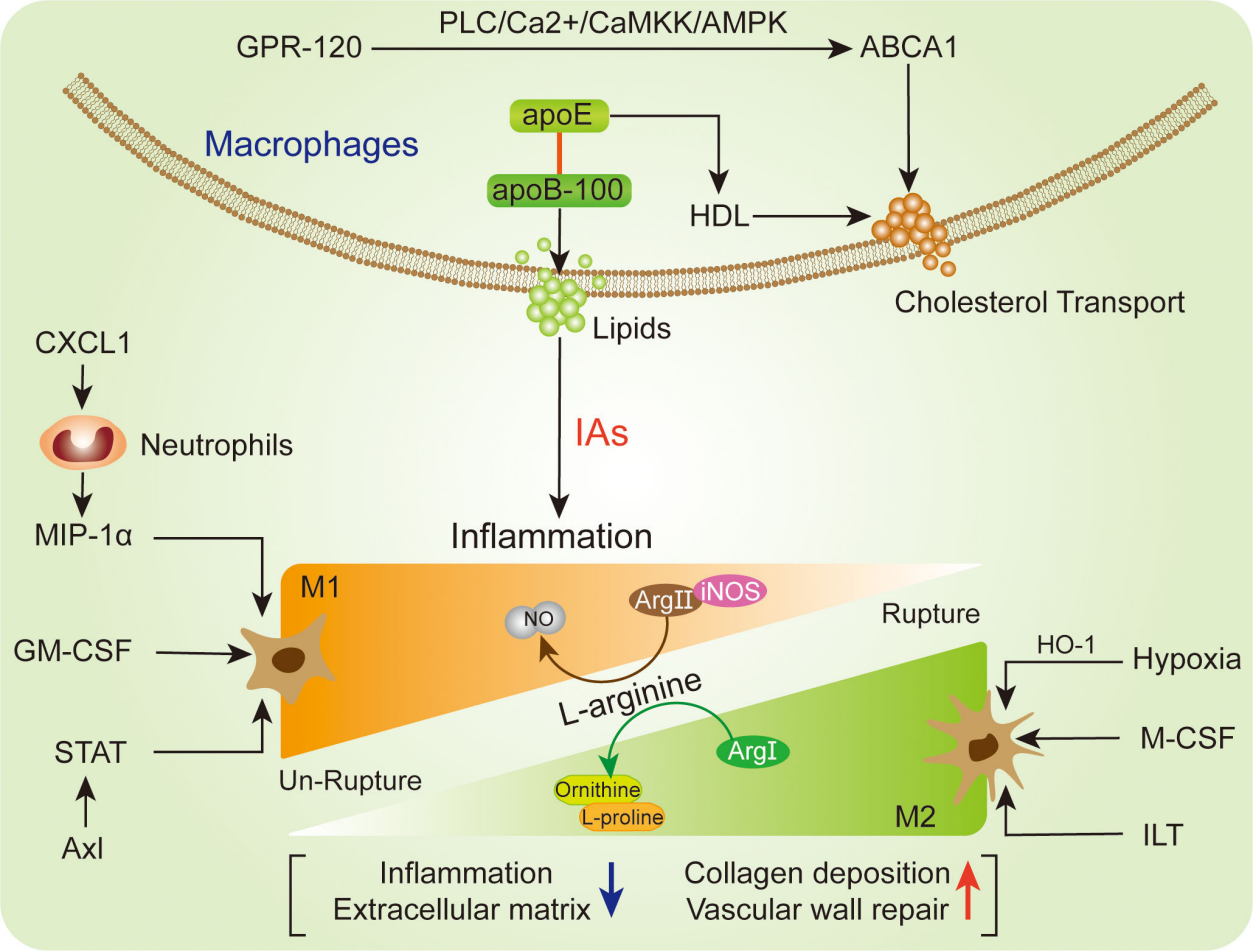
Representation of the macrophages relevant polarization (lower) and lipids regulation (upper) in IA.

Relationship between the M1 (pro-inflammatory) and M2 (reparative) macrophages subtypes will influence IA structural integrity and rupture (94). Generally, IA was characterized by mild structural alteration, and presented with M1 macrophages subtypes predominance (**Figure 3**). M1 macrophages are classical subtypes activated by IFN-α, TNF, microbial stimuli and other cytokines, which show a prevalence in the early inflammation phase (20). Recent study has demonstrated that M1 macrophages driven IA formation and growth, thereby M1/M2 ratio was increased in IA formation over time (101). In terms of L-arginine metabolic process, M1 macrophages mainly utilize arginase II (Arg II) and iNOS to increase nitric oxide (NO), while M2 macrophages transfer arginine to arginase I (Arg I) to increase ornithine and L-proline (95). Because M1 and M2 macrophage subtypes are extreme forms of a functional continuum, both of them polarization have some limitations. The M2 macrophages are alternatively activated macrophages and play an important role in clearing of extracellular matrix, vascular wall repair/remodeling and inflammation resolution (104). Understanding macrophages differentiation will decipher important pathophysiological mechanisms that occur in IA progression.

On the other hand, after IA rupture, the M2 macrophages polarization was outnumbered than M1 macrophages (105). M2 macrophages upregulation was a distinctive feature of ruptured IA with severe structural changes. The accumulation of erythrocytes and/or their degradation by-product could shift macrophages towards M2 phenotype (21). Due to compensatory regulation, the M2 macrophages potentially be elevated in the wall of ruptured IA, consequently contributing to upregulated M2/M1 ratio (16). The M2 macrophages participated in lesion fibrosis, granulations and wound healing through generating extracellular matrix, VEGF and CCL18 (106, 107). Meanwhile, M2 macrophages were also reported to promote histologic healing and collagen deposition in ruptured IA (108). In line with this, the intramural blood leakage before IA rupture potentially promotes M2 macrophages polarization, subsequently initiating aneurysm wall repair and remodeling (16). However, with the aneurysm structure deterioration and severe destructed aneurysm wall after IA, the M2 macrophages will be continuously reduced (105). One hypothesis is that intraluminal thrombus (ILT) lead to fragile aneurysm wall and macrophages polarization towards M2 phenotype, as well as enhancing bioreactivity of M2 macrophages (16, 109). For the extracellular stimulation, M-CSF supposed to be linked with M2 macrophages polarization, while above process cannot exhibit full M2 phenotypes and can be reversibly modulated by antagonistic agent (110). Due to oxidative stress cause of the programmed cell death intrinsic activation within the aneurysm wall (111), the hypoxic condition was deemed to be another stimulator for the M2 macrophages polarization (16). The M2 macrophages cellular marker CD163 and HO-1 showed a great potential in antioxidant defense and were significantly elevated in ruptured IA (112, 113). These complexities were associated with the variable inflammatory injure, stimulus exposure, and distinct cellular features of macrophages in IA.

## Characteristics of macrophage lipids regulation

The cellular metabolism is essential for macrophages function maintenance, especially lipid accumulation. The intracellular lipid accumulation disturbs the homeostasis of the lipid-laden cells (114, 115). The coordinating role between ApoB/E lipoprotein particles (VLDL and LDL) and ApoA (HDL) is the major protein fundament of lipids transportation from circulation into the vessel walls, and lipid accumulation might trigger cell death and inflammation in the IA lesion (**Figure 3**). Of note, both CD68- and CD163-positive macrophages were presented with high levels of apoB-100 and adipophilin (116). The adipophilin is a protein attached to intracellular lipid droplets and contributes to lipid accumulation, thereby reflecting the death of macrophages (117). However, the apoB-100 is mostly abundant in extracellular matrix and contributes local lipids-derived inflammation, whose accumulation is associated with IA wall degeneration and macrophages infiltration (117). Intriguingly, as a molecular containing apoB-100 structure lipoproteins, ApoE shows the ability to upregulate extracellular retention of HDL (116, 118). In the arterial intima, HDL particles are able to initiate the macrophage-specific reverse cholesterol transport related multistep pathways (119, 120). In terms of this, macrophages activation may reduce lipid burden and reverse cholesterol transport. Moreover, intracellular cholesterol efflux transporter ABCA1 expression in macrophages extent was negatively associated with lipid accumulation and macrophages infiltration in IA (116). With the stimulation of G protein-coupled receptor (GPR-120), the ABCA1-mediated free cholesterol efflux will be enhanced through PLC/Ca2^+^/CaMKK/AMPK cascade in macrophages (121). The activation of GPR-120 has been observed to suppress macrophages infiltration and inflammatory response in un-ruptured IA lesion (122). On the other hand, recent study reported that the macrophages-derived foam cells could increase extracellular cholesterol levels and facilitate degenerative changes, thereby promoting the progression of IA (123, 124). The deposition of lipid on macrophages will facilitate foam cells transformation (123). Due to the cytotoxic lipids, the activated macrophages might increase lipid burden, foam cell formation and vascular wall smooth muscle cells (SMCs) loss, thereby promoting un-ruptured IA degenerative remodeling and rupture risk (117).

## Macrophages relevant signaling pathways and therapy strategies in IA

### NF-κB signaling

**Figure 4:**
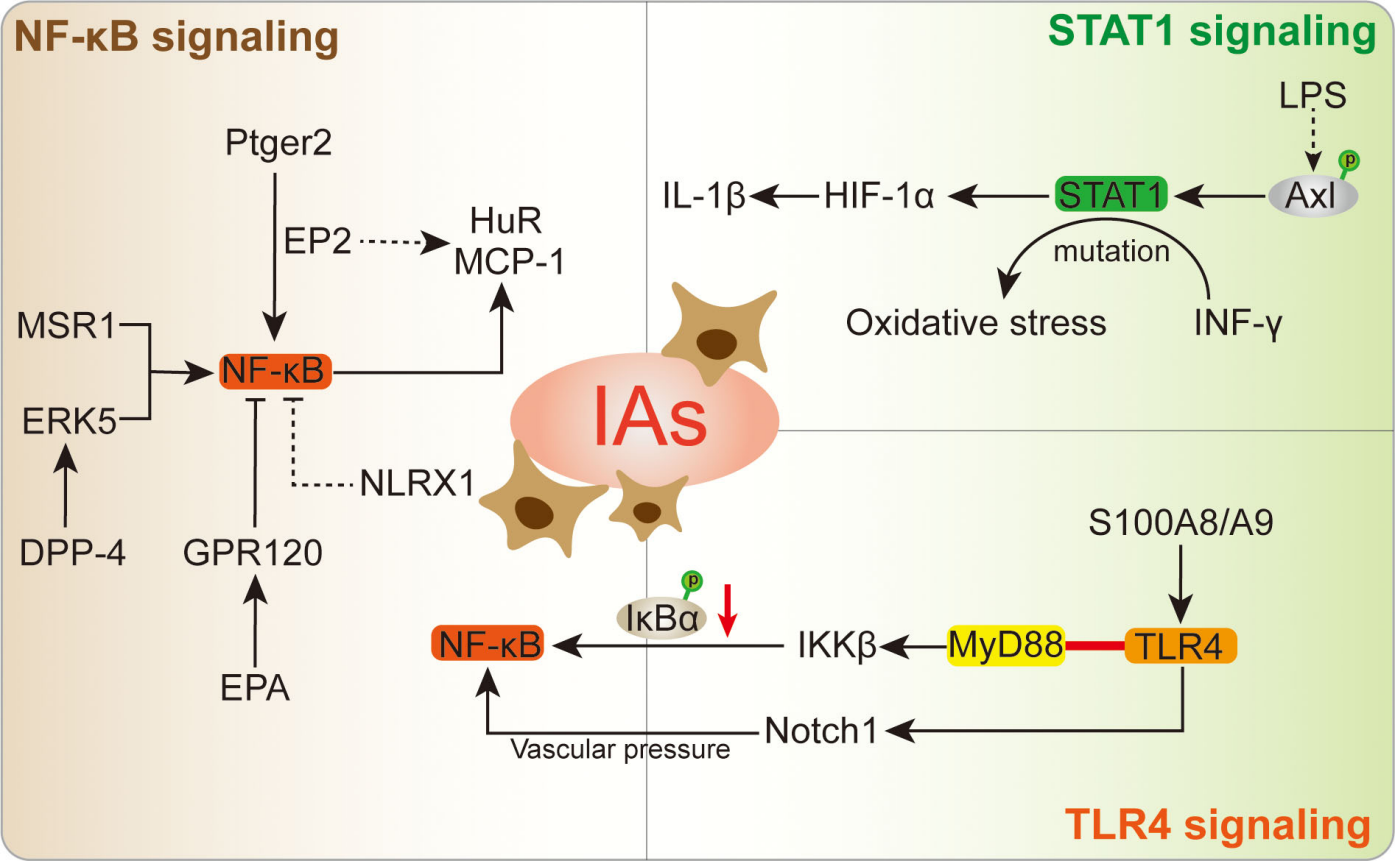
The specific role played by NF-κB, STAT1, and TLR4 signaling in macrophages following IA.

The NF-κB signaling is important for macrophages, which is a most prevalent modulator for macrophages in IA lesion (**Figure 4**). Ptger2 and IκBα genomic alteration would inhibit the nuclear translocation of NF-κB in macrophages, subsequently influencing the macrophages inflammatory responses in IA (17). The activation of NF-κB is a major transcription signaling for important pro-inflammatory genes regulation, such as TNF, IL-1β, MMPs, and COX-2. The specific deletion of Ptger2, an EP_2_ encoding protein, which will dampen the macrophages pathogenic effect like infiltration and inflammatory activation through EP_2_/NF-kB cascade (10). The above mechanism plays a crucial role in the IA progression, especially macrophages infiltration and activation (10). Of note, EP_2_ not merely stimulates NF-κB and macrophages inflammation, but also induces RNA-binding protein (HuR) to stabilize and encode MCP-1 generation (10). The administration of antagonist (PF-04418948) specific for EP_2_ might be a powerful candidate to prevent IA lesion enlargement and macrophages-related degenerative changes (10). Moreover, destroying macrophages own MCP-1 chemotactic signaling in inflammatory microenvironment could suspend self-amplification loop in the IA, thereby reducing macrophages infiltration in IA walls (125). Pharmacological inhibition suppressed macrophages infiltration and IA progression in mouse model, while there are still lack further preclinical evidences. Similarly, the Anagliptin activated ERK5 in macrophages was demonstrated to promote anti-inflammatory effects by inhibiting NF-kB signaling (**Table 1**), namely inhibitory effect to MCP-1 and IL-6 production (84). Among which, the transmembrane protein DPP-4 with large extracellular domain acts as a target for Anagliptin, which could prevent the lipopolysaccharide/NF-kB-induced macrophage proinflammatory cytokines generation through activating ERK5 in IA lesion (84). Similarly, Abekura et al. reported that GPR120 could partly suppress the lipopolysaccharide-induced NF-kB signaling activation and subsequently inhibit transcriptional induction of MCP-1 (122). The GPR120 may impair macrophages chemo-attractive abundance in IA lesions, which can be remarkably enhanced by eicosapentaenoic acid (EPA) administration. However, the EPA has various targets on inflammation beyond being a GPR120 agonist (130, 131), thus, more experimental evidence of the role of EPA in IA progression is necessary for its clinical translation. Furthermore, the NLRX1 was also observed to alleviate NF-kB signaling and macrophages infiltration in IA wall (126). The silence of NLRX1 increased NF-kB signaling and apoptosis-related genes expression. However, there are few studies evaluated NLRX1 function in the brain. On the other hand, Kong et al. indicated that MSR1-induced NF-κB signaling promotes myelin debris phagocytosis and macrophages pro-inflammatory polarization, consequently leading to neuronal apoptosis (38). MSR1 primarily mediates oxidized lipoprotein uptake and facilitates foam cell production, which was also correlated with macrophages activation and apoptotic responses in IA lesion (9). In recent, the zinc-induced protein A20 and Tanshinone IIA showed a great potential in NF-κB signaling inhibition and macrophages infiltration, thereby suppressing the inflammatory responses and IA progression (79, 127). As an essential micronutrient and antioxidant supplements, zinc potentially to be a safe intervention and/or auxiliary strategy for IA clinical treatment, especially in elderly individuals (127). Additionally, zinc increased serum estrogen and progesterone levels in ovariectomized mouse, thus the preventive effect of zinc in female mouse IA progression might stronger than male, which needs further preclinical translation investigations. Taken together, understanding and intervention of NF-κB signaling is important for macrophages-relevant IA prevention and treatment.

**Table 1:** Intervene of macrophages and associated targets in IA.

Drug	Targets	Macrophage Regulation	Contribution	References
CoQ10	NA	Infiltration and oxidative stress	Positive	(39, 40)
Iron limitation	NA	Infiltration and oxidative stress	Positive	(84)
Anagliptin	DPP-4	Anti-inflammation	Positive	(84)
EPA	GPR120	Chemo-attractive abundance	Positive	(126)
Protein A20	NF-κB	Infiltration	Positive	(79, 127)
Tanshinone IIA	NF-κB	Infiltration	Positive	(79, 127)
R428	Axl	Activation and polarization	Positive	(11)
Jakinibs	STAT1	Recruitment	Positive	(128)
2-Bromoergocryptine Mesylate	TLR4	--	--	(129)
PF-04418948	EP2	Degenerative changes	Positive	(10)

### STAT1 signaling

The STAT signaling is a key factor that regulates the activation of macrophages, which is also associated with physiological processes in cell proliferation, differentiation, and apoptosis (132). As a transcriptional regulator, the STAT1 signaling could further modulate downstream target genes by binding to promoter, as well as transducing signals from the cell membrane to the nucleus (133). The interaction between STAT1 signaling and its related modulators has been emerged as important determinants of immune and inflammatory functions (133). In IA lesion, STAT1/HIF-1α signaling was proved to promote macrophages activation and polarization, whose stimulation can be enhanced by Axl phosphorylation (11). On the contrary, the STAT1 knockdown directly abolished the Axl effects on macrophages, and emphasized the key role of Axl in STAT1 signaling. As auxiliary evidence, the lipopolysaccharide (LPS) induced macrophages were accompanied with increased Axl expression and phosphorylation, which might further promote inflammatory response in IA lesion (134). The immunofluorescence colocalization of Axl and macrophage-specific marker has shown reliable evidence in the macrophages of IA lesion. The Axl-specific inhibitor R428 showed a significant inhibit effect to the levels of pSTAT1 and HIF-1α (11), wherein the STAT1 signaling is responsible for HIF-1α expression (135), ultimately decreasing the macrophages level in the IA. In line with this, the STAT1-induced HIF-1α could further stimulate IL-1β (136), which might cause macrophages-induced inflammation in unruptured IA. Due to the complex effects of Axl in IA, R428 treatment inhibits AXL activation in LPS/IFN-g-primed THP-1 cells, but human primary M1 macrophages *in vitro* did not provide additional translational evidences (11). In inflammatory diseases, the INF-γ-induced STAT1 signaling contributed to downstream transcription targets activation and oxidative stress (137, 138). Of note, this mechanism is also observed in the pro-inflammatory macrophages of IA lesion (96). Most recent, the STAT1 gain-of-function mutation was reported to strengthen the response of INF-γ and macrophages recruitment in IA (128). The upstream inhibitors administration shows potential to interfere patients STAT1 gain-of-function and improve immune dysregulation related clinical outcomes. Together, these findings potentially provide a new therapeutic target and facilitate pharmacological treatment for IA.

### TLR4 signaling

TLR4 is known as a critical receptor for innate immunity activation relevant exogenous ligands, such as lipopolysaccharide induced macrophages (139, 140). TLR4 has cytoplasmic toll/IL-1R homologous domain that can bind to MyD88, thereby promoting inflammatory macrophages in aneurysmal walls and ruptured aneurysm (13). In terms of this, TLR4 signaling could delivery activation signal to IKKβ and result in inhibitor kappaB phosphorylation and degradation, ultimately promoting NF-κB p65/p50 heterodimer activation (13) (96). In the IA initialization, upregulated TLR4 signaling was accompanied with NF-κB activation (141, 142). Interestingly, in inflammation-driven diseases, the Notch1 signaling was involved in macrophages TLR4/IKKβ/NF-κB signaling, wherein the TLR4 stimulated the Notch1 signaling and promoted NF-κB activation (143). Due to the vascular wall pressure, the Notch1 signaling-induced cells polarization existed in IA (144). The Notch signaling interaction is an important factor for IA angiogenesis molecules, inflammation and structural fragility (145, 146). In addition, the pro-inflammatory mediator S100A8/A9 heterodimer was reported to bind to TLR4 and contributed to the macrophages activation (88). The Sandip et al. reported that 2-Bromoergocryptine Mesylate is a potential candidate drug to prevent IA rupture by targeting TLR4 receptor (129). However, as a special agent for targeting TLR4 protein active region in diabetes and Alzheimer’s disease inflammation, 2-Bromoergocryptine Mesylate still has obstacles in broadening pharmacological effects for the IA.

## Conclusions and perspectives

IA is a devastating disease with high fate death ratio, as well as limited prevention and treatment approaches. In recent years, achievements have been made in our understanding of dysregulated macrophages in IA initiation and progression, whereas detailed mechanisms remain fragmentary. The macrophages were ubiquitously detected in both ruptured and unruptured IA walls. Intriguingly, the macrophages accumulation not merely lead to endothelial degeneration and hyperactive inflammatory signals, but also show a protect effect in ruptured IA under certain conditions. Hence, we summarized the regulation mechanisms regarding the roles of macrophages that may play. Particularly, through modulating polarization phenotypes, lipids metabolism, cellular signaling and inflammation stimulation, macrophages will help to clarify the IA process and provide insights to therapeutic strategies. Severe morphological changes in IA were associated with higher numbers of pro-inflammatory macrophages activation and infiltration, wherein the polarization tendency and inflammatory cytokines generation can provide some speculative insight into the nature of IA formation and rupture. Meanwhile, compelling evidences revealed NF-κB, STAT1 and TLR4 signaling cascade have an essential role in macrophages inflammatory response during IA lesion deterioration. Noteworthy, the lipid metabolism has controversial effects in macrophages, while its function in macrophages relevant IA is not negligible. The pros and cons of macrophages immune inflammatory modulation in IA pathogenesis and progression should be further elucidated.

In general, most of the macrophages intra/inter-cellular modulation has been linked to macrophages pathogenic reaction and promotes IA lesion. The nutritional restriction and anti-inflammation treatments are mild and safe approaches for IA, but clinical evidence is still lacking. According to current knowledge, suppression of pro-inflammatory macrophages accumulation and cytokines generation through modulating key molecules is crucial in prevent IA progression. The macrophages specific signaling intervention like NF-κB and STAT1 cascades showed a great potential in ameliorating IA structural changes and inflammation condition. However, due to the controversial signaling interaction between inflammation, macrophages and complex IA pathological conditions, underlying regulation mechanisms are yet to be fully clarified. Distinguishing the mechanisms of macrophages heterogeneities and hyperactivation will facilitate our understanding of personalized rupture and unruptured IA therapeutic strategies. In this regard, it will be interesting to determine the diverse abilities of macrophages in the complex context of IA lesion and develop innovative therapeutic strategies to improve clinical outcomes.

## Author contributions

JianD: Writing – original draft, Data curation. QZ: Writing – original draft, Investigation. ZH: Data curation, Writing – review & editing. ST: Software, Visualization, Writing – review & editing. JiaD: Supervision, Writing – review & editing. WX: Conceptualization, Project administration, Supervision, Writing – review & editing.

## Glossary

**Table:**

IA	Intracranial aneurysm
HR-MRI	High-resolution magnetic resonance imaging
SAH	Subarachnoid hemorrhage
MMPs	Matrix metalloproteinases
GREM1	Morphogenetic protein Gremlin 1
S1P1	Sphigosine-1-phosphate receptor type 1
PGE	Prostaglandin E
EP2	Receptor subtype 2
CoQ10	Coenzyme Q10
IL	Interleukin
iNOS	Inducible nitric oxide synthase
PPARγ	Peroxisome proliferator-activated receptor-γ
ERK5	Extracellular signal-regulated kinase 5
TLR-4	Toll-like receptor 4
NO	Nitric oxide
Arg	Arginase
STAT	Signal transducer and activator of transcription
GM-CSF	Granulocyte-macrophage colony stimulating factor
M-CSF	Macrophage colony stimulating factor
MIP-1α	Macrophage inflammatory protein-1α
ILT	Intraluminal thrombus
VLDL	Very low density lipoprotein
HDL	High density lipoprotein
GPR-120	G protein-coupled receptor
ABCA1	ATP-binding cassette transporter A1
EPA	Eicosapentaenoic acid
LPS	Lipopolysaccharide
NF-κB	Nuclear factor kappa-B
CXCL1	C-X-C motif chemokine ligand 1
ApoE	Apolipoprotein E
OXPHOS	Oxidative phosphorylation
TRAP	Triiodothyronine receptor auxiliary protein
JNK	C-Jun N-terminal Kinase
TGF-β	Transforming growth factor beta
TNF-α	Tumor necrosis factor α
HO-1	Heme oxygenase-1
COX-2	Cytochrome c oxidase subunit II
NLRX1	NLR family member X1

## References

[1] Tawk RG Hasan TF D’Souza CE Peel JB Freeman WD. Diagnosis and treatment of unruptured intracranial aneurysms and aneurysmal subarachnoid hemorrhage. Mayo Clin Proc (2021) 96(7):1970–2000. doi: 10.1016/j.mayocp.2021.01.005 33992453

[2] Kim HJ Song HN Lee JE Kim YC Baek IY Kim YS. How cerebral vessel tortuosity affects development and recurrence of aneurysm: outer curvature versus bifurcation type. J Stroke. (2021) 23(2):213–22. doi: 10.5853/jos.2020.04399 PMC8189854 34102756

[3] Samaniego EA Roa JA Hasan D. Vessel wall imaging in intracranial aneurysms. J neurointerv Surg (2019) 11(11):1105–12. doi: 10.1136/neurintsurg-2019-014938 31337731

[4] Claassen J Park S. Spontaneous subarachnoid haemorrhage. Lancet (2022) 400(10355):846–62. doi: 10.1016/S0140-6736(22)00938-2 PMC9987649 35985353

[5] Etminan N Rinkel GJ. Unruptured intracranial aneurysms: development, rupture and preventive management. Nat Rev Neurol (2016) 12(12):699–713. doi: 10.1038/nrneurol.2016.150 27808265

[6] Hasan D Chalouhi N Jabbour P Dumont AS Kung DK Magnotta VA. Early change in ferumoxytol-enhanced magnetic resonance imaging signal suggests unstable human cerebral aneurysm: a pilot study. Stroke (2012) 43(12):3258–65. doi: 10.1161/STROKEAHA.112.673400 PMC3508354 23138441

[7] Frösen J. Smooth muscle cells and the formation, degeneration, and rupture of saccular intracranial aneurysm wall–a review of current pathophysiological knowledge. Transl Stroke Res (2014) 5(3):347–56. doi: 10.1007/s12975-014-0340-3 24683005

[8] Chang HS. Simulation of the natural history of cerebral aneurysms based on data from the International Study of Unruptured Intracranial Aneurysms. J Neurosurg (2006) 104(2):188–94. doi: 10.3171/jns.2006.104.2.188 16509491

[9] Wang X Wen D You C Tao C Ma L. Comprehensive analysis of immune cell infiltration and role of MSR1 expression in aneurysmal subarachnoid haemorrhage. Cell Prolif. (2023) 56(6):e13379. doi: 10.1111/cpr.13379 36515067 PMC10280136

[10] Aoki T Frȍsen J Fukuda M Bando K Shioi G Tsuji K. Prostaglandin E2-EP2-NF-κB signaling in macrophages as a potential therapeutic target for intracranial aneurysms. Sci Signal (2017) 10(465):eaah6037. doi: 10.1126/scisignal.aah6037 28174280

[11] Han Y Li G Zhang Z Zhang X Zhao B Yang H. Axl promotes intracranial aneurysm rupture by regulating macrophage polarization toward M1 via STAT1/HIF-1α. Front Immunol (2023) 14:1158758. doi: 10.3389/fimmu.2023.1158758 37223093 PMC10200875

[12] Ashida S Yamawaki-Ogata A Tokoro M Mutsuga M Usui A Narita Y. Administration of anti-inflammatory M2 macrophages suppresses progression of angiotensin II-induced aortic aneurysm in mice. Sci Rep (2023) 13(1):1380. doi: 10.1038/s41598-023-27412-x 36697439 PMC9877022

[13] Mitsui K Ikedo T Kamio Y Furukawa H Lawton MT Hashimoto T. TLR4 (Toll-like receptor 4) mediates the development of intracranial aneurysm rupture. Hypertension (2020) 75(2):468–76. doi: 10.1161/HYPERTENSIONAHA.118.12595 PMC7377296 31865791

[14] Aoki T Koseki H Miyata H Itoh M Kawaji H Takizawa K. RNA sequencing analysis revealed the induction of CCL3 expression in human intracranial aneurysms. Sci Rep (2019) 9(1):10387. doi: 10.1038/s41598-019-46886-2 31316152 PMC6637171

[15] Jiang Z Huang J You L Zhang J. Protective effects of BP-1-102 against intracranial aneurysms-induced impairments in mice. J Drug Targeting (2021) 29(9):974–82. doi: 10.1080/1061186X.2021.1895817 33682559

[16] Stratilová MH Koblížek M Štekláčová A Beneš V Sameš M Hejčl A. Increased macrophage M2/M1 ratio is associated with intracranial aneurysm rupture. Acta Neurochir (Wien). (2023) 165(1):177–86. doi: 10.1007/s00701-022-05418-0 36437400

[17] Shimizu K Kushamae M Mizutani T Aoki T. Intracranial aneurysm as a macrophage-mediated inflammatory disease. Neurol Med Chir (Tokyo). (2019) 59(4):126–32. doi: 10.2176/nmc.st.2018-0326 PMC6465529 30867357

[18] Kanematsu Y Kanematsu M Kurihara C Tada Y Tsou TL van Rooijen N. Critical roles of macrophages in the formation of intracranial aneurysm. Stroke (2011) 42(1):173–8. doi: 10.1161/STROKEAHA.110.590976 PMC3021554 21106959

[19] Hoh BL Rojas K Lin L Fazal HZ Hourani S Nowicki KW. Estrogen deficiency promotes cerebral aneurysm rupture by upregulation of th17 cells and interleukin-17A which downregulates E-cadherin. J Am Heart Assoc (2018) 7(8):e008863. doi: 10.1161/JAHA.118.008863 29654199 PMC6015422

[20] Muhammad S Chaudhry SR Dobreva G Lawton MT Niemelä M Hänggi D. Vascular macrophages as therapeutic targets to treat intracranial aneurysms. Front Immunol (2021) 12:630381. doi: 10.3389/fimmu.2021.630381 33763073 PMC7982735

[21] Ollikainen E Tulamo R Kaitainen S Honkanen P Lehti S Liimatainen T. Macrophage infiltration in the saccular intracranial aneurysm wall as a response to locally lysed erythrocytes that promote degeneration. J Neuropathol Exp Neurol (2018) 77(10):890–903. doi: 10.1093/jnen/nly068 30113655

[22] Ollikainen E Tulamo R Frösen J Lehti S Honkanen P Hernesniemi J. Mast cells, neovascularization, and microhemorrhages are associated with saccular intracranial artery aneurysm wall remodeling. J Neuropathol Exp Neurol (2014) 73(9):855–64. doi: 10.1097/NEN.0000000000000105 25101705

[23] Wen D Wang X Chen R Li H Zheng J Fu W. Single-cell RNA sequencing reveals the pathogenic relevance of intracranial atherosclerosis in blood blister-like aneurysms. Front Immunol (2022) 13:927125. doi: 10.3389/fimmu.2022.927125 35874788 PMC9304558

[24] Pauli J Reisenauer T Winski G Sachs N Chernogubova E Freytag H. Apolipoprotein E (ApoE) rescues the contractile smooth muscle cell phenotype in popliteal artery aneurysm disease. Biomolecules (2023) 13(7):1074. doi: 10.3390/biom13071074 37509110 PMC10377618

[25] Mulorz J Spin JM Beck HC Tha Thi ML Wagenhäuser MU Rasmussen LM. Hyperlipidemia does not affect development of elastase-induced abdominal aortic aneurysm in mice. Atherosclerosis (2020) 311:73–83. doi: 10.1016/j.atherosclerosis.2020.08.012 32949946

[26] Rasmussen KL Luo J Nordestgaard BG Tybjærg-Hansen A Frikke-Schmidt R. APOE and vascular disease: Sequencing and genotyping in general population cohorts. Atherosclerosis (2023) 385:117218. doi: 10.1016/j.atherosclerosis.2023.117218 37586954

[27] Martinez AN Tortelote GG Pascale CL McCormack IG Nordham KD Suder NJ. Single-cell transcriptome analysis of the circle of willis in a mouse cerebral aneurysm model. Stroke (2022) 53(8):2647–57. doi: 10.1161/STROKEAHA.122.038776 35770669

[28] Zhong A Wang F Zhou Y Ding N Yang G Chai X. Molecular subtypes and machine learning-based predictive models for intracranial aneurysm rupture. World Neurosurg (2023). 179:e166-e186. doi: 10.1016/j.wneu.2023.08.043 37597661

[29] Feng Z Zhang X Li L Wang C Feng M Zhao K. Tumor-associated macrophage-derived exosomal microRNA-155-5p stimulates intracranial aneurysm formation and macrophage infiltration. Clin Sci (Lond). (2019) 133(22):2265–82. doi: 10.1042/CS20190680 31657855

[30] Yamamoto R Aoki T Koseki H Fukuda M Hirose J Tsuji K. A sphingosine-1-phosphate receptor type 1 agonist, ASP4058, suppresses intracranial aneurysm through promoting endothelial integrity and blocking macrophage transmigration. Br J Pharmacol (2017) 174(13):2085–101. doi: 10.1111/bph.13820 PMC5466536 28409823

[31] Galvani S Sanson M Blaho VA Swendeman SL Obinata H Conger H. HDL-bound sphingosine 1-phosphate acts as a biased agonist for the endothelial cell receptor S1P1 to limit vascular inflammation. Sci Signal (2015) 8(389):ra79. doi: 10.1126/scisignal.aaa2581 26268607 PMC4768813

[32] Nitzsche A Poittevin M Benarab A Bonnin P Faraco G Uchida H. Endothelial S1P(1) signaling counteracts infarct expansion in ischemic stroke. Circ Res (2021) 128(3):363–82. doi: 10.1161/CIRCRESAHA.120.316711 PMC7874503 33301355

[33] Qu C Mao C Xiao P Shen Q Zhong YN Yang F. Ligand recognition, unconventional activation, and G protein coupling of the prostaglandin E(2) receptor EP2 subtype. Sci Adv (2021) 7(14):eabf1268. doi: 10.1126/sciadv.abf1268 33811074 PMC11057787

[34] Avendaño MS García-Redondo AB Zalba G González-Amor M Aguado A Martínez-Revelles S. mPGES-1 (Microsomal prostaglandin E synthase-1) mediates vascular dysfunction in hypertension through oxidative stress. Hypertension (2018) 72(2):492–502. doi: 10.1161/HYPERTENSIONAHA.118.10833 29891646

[35] Yau L Zahradka P. PGE(2) stimulates vascular smooth muscle cell proliferation via the EP2 receptor. Mol Cell Endocrinol (2003) 203(1-2):77–90. doi: 10.1016/S0303-7207(03)00096-0 12782405

[36] Wang M Wang Y Xie T Zhan P Zou J Nie X. Prostaglandin E(2)/EP(2) receptor signalling pathway promotes diabetic retinopathy in a rat model of diabetes. Diabetologia (2019) 62(2):335–48. doi: 10.1007/s00125-018-4755-3 30411254

[37] Gudgeon J Marín-Rubio JL Trost M. The role of macrophage scavenger receptor 1 (MSR1) in inflammatory disorders and cancer. Front Immunol (2022) 13:1012002. doi: 10.3389/fimmu.2022.1012002 36325338 PMC9618966

[38] Kong FQ Zhao SJ Sun P Liu H Jie J Xu T. Macrophage MSR1 promotes the formation of foamy macrophage and neuronal apoptosis after spinal cord injury. J Neuroinflammation. (2020) 17(1):62. doi: 10.1186/s12974-020-01735-2 32066456 PMC7027125

[39] Huang J Zhang H You L Zhang J Jiang Z. Coenzyme Q10 inhibits intracranial aneurysm formation and progression in a mouse model. Pediatr Res (2022) 91(4):839–45. doi: 10.1038/s41390-021-01512-8 33859365

[40] Kawakatsu T Kamio Y Makino H Hokamura K Imai R Sugimura S. Dietary iron restriction protects against aneurysm rupture in a mouse model of intracranial aneurysm. Cerebrovasc Dis (2023). doi: 10.1159/000531431. [Epub ahead of print]. PMC12407094 37290410

[41] Shimizu K Kushamae M Aoki T. Macrophage imaging of intracranial aneurysms. Neurol Med Chir (Tokyo). (2019) 59(7):257–63. doi: 10.2176/nmc.st.2019-0034 PMC6635145 31080227

[42] Marasini A Shrestha A Phuyal S Zaidat OO Kalia JS. Role of artificial intelligence in unruptured intracranial aneurysm: an overview. Front Neurol (2022) 13:784326. doi: 10.3389/fneur.2022.784326 35280303 PMC8904392

[43] Morel S Diagbouga MR Dupuy N Sutter E Braunersreuther V Pelli G. Correlating clinical risk factors and histological features in ruptured and unruptured human intracranial aneurysms: the swiss aneuX study. J Neuropathol Exp Neurol (2018) 77(7):555–66. doi: 10.1093/jnen/nly031 PMC6005054 29688417

[44] Sawyer DM Pace LA Pascale CL Kutchin AC O’Neill BE Starke RM. Lymphocytes influence intracranial aneurysm formation and rupture: role of extracellular matrix remodeling and phenotypic modulation of vascular smooth muscle cells. J Neuroinflammation. (2016) 13(1):185. doi: 10.1186/s12974-016-0654-z 27416931 PMC4946206

[45] Aoki T Saito M Koseki H Tsuji K Tsuji A Murata K. Macrophage imaging of cerebral aneurysms with ferumoxytol: an exploratory study in an animal model and in patients. J Stroke Cerebrovasc Dis (2017) 26(10):2055–64. doi: 10.1016/j.jstrokecerebrovasdis.2016.10.026 28774792

[46] Furukawa H Wada K Tada Y Kuwabara A Sato H Ai J. Mast cell promotes the development of intracranial aneurysm rupture. Stroke (2020) 51(11):3332–9. doi: 10.1161/STROKEAHA.120.030834 PMC7606717 33019897

[47] Ge P Liu C Chan L Pang Y Li H Zhang Q. High-dimensional immune profiling by mass cytometry revealed the circulating immune cell landscape in patients with intracranial aneurysm. Front Immunol (2022) 13:922000. doi: 10.3389/fimmu.2022.922000 35833148 PMC9271834

[48] Koch AE Haines GK Rizzo RJ Radosevich JA Pope RM Robinson PG. Human abdominal aortic aneurysms. Immunophenotypic analysis suggesting an immune-mediated response. Am J Pathol (1990) 137(5):1199–213. PMC1877681 1700620

[49] Lindholt JS Shi GP. Chronic inflammation, immune response, and infection in abdominal aortic aneurysms. Eur J Vasc Endovasc Surg (2006) 31(5):453–63. doi: 10.1016/j.ejvs.2005.10.030 16414293

[50] Saburi M Yamada H Wada N Motoyama S Sugimoto T Kubota H. Maternal high-fat diet promotes abdominal aortic aneurysm expansion in adult offspring by epigenetic regulation of IRF8-mediated osteoclast-like macrophage differentiation. Cells (2021) 10(9):2224. doi: 10.3390/cells10092224 34571873 PMC8466477

[51] Takei Y Tanaka T Kent KC Yamanouchi D. Osteoclastogenic differentiation of macrophages in the development of abdominal aortic aneurysms. Arterioscler Thromb Vasc Biol (2016) 36(9):1962–71. doi: 10.1161/ATVBAHA.116.307715 27386936

[52] Yuan Z Lu Y Wei J Wu J Yang J Cai Z. Abdominal aortic aneurysm: roles of inflammatory cells. Front Immunol (2020) 11:609161. doi: 10.3389/fimmu.2020.609161 33613530 PMC7886696

[53] Wang Y Jia L Xie Y Cai Z Liu Z Shen J. Involvement of macrophage-derived exosomes in abdominal aortic aneurysms development. Atherosclerosis (2019) 289:64–72. doi: 10.1016/j.atherosclerosis.2019.08.016 31479773

[54] Raffort J Lareyre F Clément M Hassen-Khodja R Chinetti G Mallat Z. Monocytes and macrophages in abdominal aortic aneurysm. Nat Rev Cardiol (2017) 14(8):457–71. doi: 10.1038/nrcardio.2017.52 28406184

[55] Police SB Thatcher SE Charnigo R Daugherty A Cassis LA. Obesity promotes inflammation in periaortic adipose tissue and angiotensin II-induced abdominal aortic aneurysm formation. Arterioscler Thromb Vasc Biol (2009) 29(10):1458–64. doi: 10.1161/ATVBAHA.109.192658 PMC2753598 19608970

[56] Williams H Wadey KS Frankow A Blythe HC Forbes T Johnson JL. Aneurysm severity is suppressed by deletion of CCN4. J Cell Commun Signal (2021) 15(3):421–32. doi: 10.1007/s12079-021-00623-5 PMC8222476 34080128

[57] Baranyi U Stern C Winter B Türkcan A Scharinger B Stelzmüller ME. The megaaortic syndrome: Progression of ascending aortic aneurysm or a disease of distinct origin? Int J Cardiol (2017) 227:717–26. doi: 10.1016/j.ijcard.2016.10.072 27836291

[58] Ren P Zhang L Xu G Palmero LC Albini PT Coselli JS. ADAMTS-1 and ADAMTS-4 levels are elevated in thoracic aortic aneurysms and dissections. Ann Thorac Surg (2013) 95(2):570–7. doi: 10.1016/j.athoracsur.2012.10.084 PMC3593585 23245439

[59] Johnston WF Salmon M Pope NH Meher A Su G Stone ML. Inhibition of interleukin-1β decreases aneurysm formation and progression in a novel model of thoracic aortic aneurysms. Circulation (2014) 130(11 Suppl 1):S51–9. doi: 10.1161/CIRCULATIONAHA.113.006800 PMC5097450 25200056

[60] Ren P Wu D Appel R Zhang L Zhang C Luo W. Targeting the NLRP3 inflammasome with inhibitor MCC950 prevents aortic aneurysms and dissections in mice. J Am Heart Assoc (2020) 9(7):e014044. doi: 10.1161/JAHA.119.014044 32223388 PMC7428617

[61] Di Gregoli K Mohamad Anuar NN Bianco R White SJ Newby AC George SJ. MicroRNA-181b controls atherosclerosis and aneurysms through regulation of TIMP-3 and elastin. Circ Res (2017) 120(1):49–65. doi: 10.1161/CIRCRESAHA.116.309321 27756793 PMC5214094

[62] Xia L Sun C Zhu H Zhai M Zhang L Jiang L. Melatonin protects against thoracic aortic aneurysm and dissection through SIRT1-dependent regulation of oxidative stress and vascular smooth muscle cell loss. J Pineal Res (2020) 69(1):e12661. doi: 10.1111/jpi.12661 32329099

[63] Ostberg NP Zafar MA Ziganshin BA Elefteriades JA. The genetics of thoracic aortic aneurysms and dissection: A clinical perspective. Biomolecules (2020) 10(2):182. doi: 10.3390/biom10020182 31991693 PMC7072177

[64] Wortmann M Peters AS Erhart P Körfer D Böckler D Dihlmann S. Inflammasomes in the pathophysiology of aortic disease. Cells (2021) 10(9):2433. doi: 10.3390/cells10092433 34572082 PMC8468335

[65] Wang X Zhang H Cao L He Y Ma A Guo W. The role of macrophages in aortic dissection. Front Physiol (2020) 11:54. doi: 10.3389/fphys.2020.00054 32116765 PMC7013038

[66] Karlow SL McCool-Myers M Henn MC Sheth AN Owens S Kottke MJ. Trends in chlamydia and gonorrhea testing and positivity rates in a safety net hospital in Georgia: 2014 to 2017. Sex Transm Dis (2022) 49(1):29–37. doi: 10.1097/OLQ.0000000000001522 34310527

[67] Ren P Hughes M Krishnamoorthy S Zou S Zhang L Wu D. Critical role of ADAMTS-4 in the development of sporadic aortic aneurysm and dissection in mice. Sci Rep (2017) 7(1):12351. doi: 10.1038/s41598-017-12248-z 28955046 PMC5617887

[68] Wang T He X Liu X Liu Y Zhang W Huang Q. Weighted gene co-expression network analysis identifies FKBP11 as a key regulator in acute aortic dissection through a NF-kB dependent pathway. Front Physiol (2017) 8:1010. doi: 10.3389/fphys.2017.01010 29255427 PMC5723018

[69] Wang Y Yin P Chen YH Yu YS Ye WX Huang HY. A functional variant of SMAD4 enhances macrophage recruitment and inflammatory response via TGF-β signal activation in Thoracic aortic aneurysm and dissection. Aging (Albany NY). (2018) 10(12):3683–701. doi: 10.18632/aging.101662 PMC6326647 30530919

[70] Ju X Ijaz T Sun H Ray S Lejeune W Lee C. Interleukin-6-signal transducer and activator of transcription-3 signaling mediates aortic dissections induced by angiotensin II via the T-helper lymphocyte 17-interleukin 17 axis in C57BL/6 mice. Arterioscler Thromb Vasc Biol (2013) 33(7):1612–21. doi: 10.1161/ATVBAHA.112.301049 PMC3818154 23685554

[71] Khayat AA Alkhaldi AJ. Neonatal Lupus presenting with neonatal hemochromatosis-like liver disease that responded to steroids: a case report. BMC Pediatr (2022) 22(1):630. doi: 10.1186/s12887-022-03713-4 36329404 PMC9632081

[72] Adachi Y Ueda K Nomura S Ito K Katoh M Katagiri M. Beiging of perivascular adipose tissue regulates its inflammation and vascular remodeling. Nat Commun (2022) 13(1):5117. doi: 10.1038/s41467-022-32658-6 36071032 PMC9452496

[73] Yang H Yang F Luo M Chen Q Liu X Zhang Y. Metabolomic profile reveals that ceramide metabolic disturbance plays an important role in thoracic aortic dissection. Front Cardiovasc Med (2022) 9:826861. doi: 10.3389/fcvm.2022.826861 35211530 PMC8861291

[74] Andreata F Syvannarath V Clement M Delbosc S Guedj K Fornasa G. Macrophage CD31 signaling in dissecting aortic aneurysm. J Am Coll Cardiol (2018) 72(1):45–57. doi: 10.1016/j.jacc.2018.04.047 29957231

[75] Son BK Sawaki D Tomida S Fujita D Aizawa K Aoki H. Granulocyte macrophage colony-stimulating factor is required for aortic dissection/intramural haematoma. Nat Commun (2015) 6:6994. doi: 10.1038/ncomms7994 25923510

[76] Yin ZQ Han H Yan X Zheng QJ. Research progress on the pathogenesis of aortic dissection. Curr Probl Cardiol (2023) 48(8):101249. doi: 10.1016/j.cpcardiol.2022.101249 35568084

[77] Lian G Li X Zhang L Zhang Y Sun L Zhang X. Macrophage metabolic reprogramming aggravates aortic dissection through the HIF1α-ADAM17 pathway(✰). EBioMedicine (2019) 49:291–304. doi: 10.1016/j.ebiom.2019.09.041 31640947 PMC6945268

[78] Suzuki H Mikami T Tamada T Ukai R Akiyama Y Yamamura A. Inflammation promotes progression of thrombi in intracranial thrombotic aneurysms. Neurosurg Rev (2020) 43(6):1565–73. doi: 10.1007/s10143-019-01184-3 31686254

[79] Ma J Hou D Wei Z Zhu J Lu H Li Z. Tanshinone IIA attenuates cerebral aneurysm formation by inhibiting the NF−κB−mediated inflammatory response. Mol Med Rep (2019) 20(2):1621–8. doi: 10.3892/mmr.2019.10407 PMC6625418 31257487

[80] Aoki T Yamamoto K Fukuda M Shimogonya Y Fukuda S Narumiya S. Sustained expression of MCP-1 by low wall shear stress loading concomitant with turbulent flow on endothelial cells of intracranial aneurysm. Acta Neuropathol Commun (2016) 4(1):48. doi: 10.1186/s40478-016-0318-3 27160403 PMC4862234

[81] Frösen J Cebral J Robertson AM Aoki T. Flow-induced, inflammation-mediated arterial wall remodeling in the formation and progression of intracranial aneurysms. Neurosurg Focus (2019) 47(1):E21. doi: 10.3171/2019.5.FOCUS19234 PMC7193287 31261126

[82] Moriwaki T Takagi Y Sadamasa N Aoki T Nozaki K Hashimoto N. Impaired progression of cerebral aneurysms in interleukin-1beta-deficient mice. Stroke (2006) 37(3):900–5. doi: 10.1161/01.STR.0000204028.39783.d9 16439700

[83] Shimada K Furukawa H Wada K Korai M Wei Y Tada Y. Protective role of peroxisome proliferator-activated receptor-γ in the development of intracranial aneurysm rupture. Stroke (2015) 46(6):1664–72. doi: 10.1161/STROKEAHA.114.007722 PMC4447500 25931465

[84] Ikedo T Minami M Kataoka H Hayashi K Nagata M Fujikawa R. Dipeptidyl peptidase-4 inhibitor anagliptin prevents intracranial aneurysm growth by suppressing macrophage infiltration and activation. J Am Heart Assoc (2017) 6(6):e004777. doi: 10.1161/JAHA.116.004777 28630262 PMC5669147

[85] Kamińska J Tylicka M Dymicka-Piekarska V Mariak Z Matowicka-Karna J Koper-Lenkiewicz OM. Canonical NF-κB signaling pathway and GRO-α/CXCR2 axis are activated in unruptured intracranial aneurysm patients. Sci Rep (2022) 12(1):21375. doi: 10.1038/s41598-022-25855-2 36494512 PMC9734124

[86] SunBLiuZYuZ. miRNA-323a-3p promoted intracranial, aneurysm-induced inflammation via AMPK/NF-κB signaling pathway by AdipoR1. Adv Clin Exp Med (2022) 31(11):1243–54. doi: 10.17219/acem/151053 36047894

[87] Lai XL Deng ZF Zhu XG Chen ZH. Apc gene suppresses intracranial aneurysm formation and rupture through inhibiting the NF-κB signaling pathway mediated inflammatory response. Biosci Rep (2019) 39(3):BSR20181909. doi: 10.1042/BSR20181909 30808715 PMC6434386

[88] de Korte AM Aquarius R Vogl T Roth J Bartels R Boogaarts HD. Elevation of inflammatory S100A8/S100A9 complexes in intracranial aneurysms. J neurointerv Surg (2020) 12(11):1117–21. doi: 10.1136/neurintsurg-2019-015753 32332055

[89] Li Y Chen B Yang X Zhang C Jiao Y Li P. S100a8/a9 signaling causes mitochondrial dysfunction and cardiomyocyte death in response to ischemic/reperfusion injury. Circulation (2019) 140(9):751–64. doi: 10.1161/CIRCULATIONAHA.118.039262 31220942

[90] Flynn MC Kraakman MJ Tikellis C Lee MKS Hanssen NMJ Kammoun HL. Transient intermittent hyperglycemia accelerates atherosclerosis by promoting myelopoiesis. Circ Res (2020) 127(7):877–92. doi: 10.1161/CIRCRESAHA.120.316653 PMC7486277 32564710

[91] Vogl T Stratis A Wixler V Völler T Thurainayagam S Jorch SK. Autoinhibitory regulation of S100A8/S100A9 alarmin activity locally restricts sterile inflammation. J Clin Invest. (2018) 128(5):1852–66. doi: 10.1172/JCI89867 PMC5919817 29611822

[92] Vogl T Eisenblätter M Völler T Zenker S Hermann S van Lent P. Alarmin S100A8/S100A9 as a biomarker for molecular imaging of local inflammatory activity. Nat Commun (2014) 5:4593. doi: 10.1038/ncomms5593 25098555 PMC4143994

[93] Fernández-Regueras M Carbonell C Salete-Granado D García JL Gragera M Pérez-Nieto M. Predominantly pro-inflammatory phenotype with mixed M1/M2 polarization of peripheral blood classical monocytes and monocyte-derived macrophages among patients with excessive ethanol intake. Antioxidants (Basel) (2023) 12(9):1708. doi: 10.3390/antiox12091708 37760011 PMC10525853

[94] Hasan D Chalouhi N Jabbour P Hashimoto T. Macrophage imbalance (M1 vs. M2) and upregulation of mast cells in wall of ruptured human cerebral aneurysms: preliminary results. J Neuroinflammation. (2012) 9:222. doi: 10.1186/1742-2094-9-222 22999528 PMC3488554

[95] Weisser SB McLarren KW Kuroda E Sly LM. Generation and characterization of murine alternatively activated macrophages. Methods Mol Biol (2013) 946:225–39. doi: 10.1007/978-1-62703-128-8_14 23179835

[96] Shao L Qin X Liu J Jian Z Xiong X Liu R. Macrophage polarization in cerebral aneurysm: perspectives and potential targets. J Immunol Res (2017) 2017:8160589. doi: 10.1155/2017/8160589 29445758 PMC5763122

[97] Chalouhi N Theofanis T Starke RM Zanaty M Jabbour P Dooley SA. Potential role of granulocyte-monocyte colony-stimulating factor in the progression of intracranial aneurysms. DNA Cell Biol (2015) 34(1):78–81. doi: 10.1089/dna.2014.2618 25389911 PMC4281873

[98] Li H Bai S Ao Q Wang X Tian X Li X. Modulation of immune-inflammatory responses in abdominal aortic aneurysm: emerging molecular targets. J Immunol Res (2018) 2018:7213760. doi: 10.1155/2018/7213760 29967801 PMC6008668

[99] Chen B Xie K Zhang J Yang L Zhou H Zhang L. Comprehensive analysis of mitochondrial dysfunction and necroptosis in intracranial aneurysms from the perspective of predictive, preventative, and personalized medicine. Apoptosis (2023) 28(9-10):1452–68. doi: 10.1007/s10495-023-01865-x PMC10425526 37410216

[100] Sica A Mantovani A. Macrophage plasticity and polarization: in *vivo* veritas. J Clin Invest. (2012) 122(3):787–95. doi: 10.1172/JCI59643 PMC3287223 22378047

[101] Nowicki KW Hosaka K Walch FJ Scott EW Hoh BL. M1 macrophages are required for murine cerebral aneurysm formation. J neurointerv Surg (2018) 10(1):93–7. doi: 10.1136/neurintsurg-2016-012911 PMC7814362 28196918

[102] Heymann F Hammerich L Storch D Bartneck M Huss S Rüsseler V. Hepatic macrophage migration and differentiation critical for liver fibrosis is mediated by the chemokine receptor C-C motif chemokine receptor 8 in mice. Hepatology (2012) 55(3):898–909. doi: 10.1002/hep.24764 22031018 PMC4533854

[103] Shi H Han X Sun Y Shang C Wei M Ba X. Chemokine (C-X-C motif) ligand 1 and CXCL2 produced by tumor promote the generation of monocytic myeloid-derived suppressor cells. Cancer Sci (2018) 109(12):3826–39. doi: 10.1111/cas.13809 PMC6272093 30259595

[104] Khashim Z Daying D Hong DY Ringler JA Herting S Jakaitis D. The distribution and role of M1 and M2 macrophages in aneurysm healing after platinum coil embolization. AJNR Am J Neuroradiol (2020) 41(9):1657–62. doi: 10.3174/ajnr.A6719 PMC7583121 32816763

[105] Wen D Chen R Li H Zheng J Fu W Shi Z. Reduced M2 macrophages and adventitia collagen dampen the structural integrity of blood blister-like aneurysms and induce preoperative rerupture. Cell Prolif. (2022) 55(2):e13175. doi: 10.1111/cpr.13175 34970805 PMC8828257

[106] Liu W Yu M Xie D Wang L Ye C Zhu Q. Melatonin-stimulated MSC-derived exosomes improve diabetic wound healing through regulating macrophage M1 and M2 polarization by targeting the PTEN/AKT pathway. Stem Cell Res Ther (2020) 11(1):259. doi: 10.1186/s13287-020-01756-x 32600435 PMC7322868

[107] He C Yang Z Jin Y Qi X Chu J Deng X. ADM scaffolds generate a pro-regenerative microenvironment during full-thickness cutaneous wound healing through M2 macrophage polarization via lamtor1. Front Physiol (2018) 9:657. doi: 10.3389/fphys.2018.00657 29915541 PMC5994424

[108] Chau SM Herting SM Noltensmeyer DA Ahmed H Maitland DJ Raghavan S. Macrophage activation in response to shape memory polymer foam-coated aneurysm occlusion devices. J BioMed Mater Res B Appl Biomater. (2022) 110(7):1535–44. doi: 10.1002/jbm.b.35015 PMC9106830 35090200

[109] Haider P Kral-Pointner JB Mayer J Richter M Kaun C Brostjan C. Neutrophil extracellular trap degradation by differently polarized macrophage subsets. Arterioscler Thromb Vasc Biol (2020) 40(9):2265–78. doi: 10.1161/ATVBAHA.120.314883 PMC7447175 32673525

[110] Hamilton TA Zhao C Pavicic PG Jr. Datta S. Myeloid colony-stimulating factors as regulators of macrophage polarization. Front Immunol (2014) 5:554. doi: 10.3389/fimmu.2014.00554 25484881 PMC4240161

[111] Laaksamo E Tulamo R Liiman A Baumann M Friedlander RM Hernesniemi J. Oxidative stress is associated with cell death, wall degradation, and increased risk of rupture of the intracranial aneurysm wall. Neurosurgery (2013) 72(1):109–17. doi: 10.1227/NEU.0b013e3182770e8c 23096423

[112] Kurki MI Häkkinen SK Frösen J Tulamo R von und zu Fraunberg M Wong G. Upregulated signaling pathways in ruptured human saccular intracranial aneurysm wall: an emerging regulative role of Toll-like receptor signaling and nuclear factor-κB, hypoxia-inducible factor-1A, and ETS transcription factors. Neurosurgery (2011) 68(6):1667–75. doi: 10.1227/NEU.0b013e318210f001 21336216

[113] Mazur-Bialy AI Pocheć E. The time-course of antioxidant irisin activity: role of the nrf2/HO-1/HMGB1 axis. Antioxidants (Basel). (2021) 10(1):88. doi: 10.3390/antiox10010088 33440644 PMC7827448

[114] Morgan PK Huynh K Pernes G Miotto PM Mellett NA Giles C. Macrophage polarization state affects lipid composition and the channeling of exogenous fatty acids into endogenous lipid pools. J Biol Chem (2021) 297(6):101341. doi: 10.1016/j.jbc.2021.101341 34695418 PMC8604758

[115] Lee J Choi JH. Deciphering macrophage phenotypes upon lipid uptake and atherosclerosis. Immune Netw (2020) 20(3):e22. doi: 10.4110/in.2020.20.e22 32655970 PMC7327152

[116] Ollikainen E Tulamo R Lehti S Lee-Rueckert M Hernesniemi J Niemelä M. Smooth muscle cell foam cell formation, apolipoproteins, and ABCA1 in intracranial aneurysms: implications for lipid accumulation as a promoter of aneurysm wall rupture. J Neuropathol Exp Neurol (2016) 75(7):689–99. doi: 10.1093/jnen/nlw041 PMC4913436 27283327

[117] Frösen J Tulamo R Heikura T Sammalkorpi S Niemelä M Hernesniemi J. Lipid accumulation, lipid oxidation, and low plasma levels of acquired antibodies against oxidized lipids associate with degeneration and rupture of the intracranial aneurysm wall. Acta Neuropathol Commun (2013) 1:71. doi: 10.1186/2051-5960-1-71 24252658 PMC3893371

[118] Wiśniewska A Olszanecki R Totoń-Żurańska J Kuś K Stachowicz A Suski M. Anti-atherosclerotic action of agmatine in apoE-knockout mice. Int J Mol Sci (2017) 18(8):1706. doi: 10.3390/ijms18081706 28777310 PMC5578096

[119] Ito F. Polyphenols can potentially prevent atherosclerosis and cardiovascular disease by modulating macrophage cholesterol metabolism. Curr Mol Pharmacol (2021) 14(2):175–90. doi: 10.2174/1874467213666200320153410 32196455

[120] Martínez-López D Cedó L Metso J Burillo E García-León A Canyelles M. (High-density lipoprotein)-mediated macrophage cholesterol efflux in patients with abdominal aortic aneurysm-brief report. Arterioscler Thromb Vasc Biol (2018) 38(11):2750–4. doi: 10.1161/ATVBAHA.118.311704 30354236

[121] An T Zhang X Li H Dou L Huang X Man Y. GPR120 facilitates cholesterol efflux in macrophages through activation of AMPK signaling pathway. FEBS J (2020) 287(23):5080–95. doi: 10.1111/febs.15310 32243091

[122] Abekura Y Ono I Kawashima A Takizawa K Koseki H Miyata H. Eicosapentaenoic acid prevents the progression of intracranial aneurysms in rats. J Neuroinflammation. (2020) 17(1):129. doi: 10.1186/s12974-020-01802-8 32331514 PMC7181479

[123] Shimizu K Miyata H Abekura Y Oka M Kushamae M Kawamata T. High-fat diet intake promotes the enlargement and degenerative changes in the media of intracranial aneurysms in rats. J Neuropathol Exp Neurol (2019) 78(9):798–807. doi: 10.1093/jnen/nlz057 31340038

[124] Patel KM Strong A Tohyama J Jin X Morales CR Billheimer J. Macrophage sortilin promotes LDL uptake, foam cell formation, and atherosclerosis. Circ Res (2015) 116(5):789–96. doi: 10.1161/CIRCRESAHA.116.305811 PMC4602371 25593281

[125] Fukuda M Aoki T. Molecular basis for intracranial aneurysm formation. Acta Neurochir Suppl. (2015) 120:13–5. doi: 10.1007/978-3-319-04981-6_2 25366592

[126] Theus MH Brickler T Meza AL Coutermarsh-Ott S Hazy A Gris D. Loss of NLRX1 exacerbates neural tissue damage and NF-κB signaling following brain injury. J Immunol (2017) 199(10):3547–58. doi: 10.4049/jimmunol.1700251 PMC5683102 28993512

[127] Hayashi K Kataoka H Minami M Ikedo T Miyata T Shimizu K. Association of zinc administration with growth suppression of intracranial aneurysms via induction of A20. J Neurosurg (2020) 134(3):992–8. doi: 10.3171/2020.1.JNS192047 32217803

[128] Bierman-Chow S Freeman AF Holland SM Lynch J Cho HJ. Cerebral aneurysm in three pediatric patients with STAT1 gain-of-function mutations. J Neurol (2022) 269(10):5638–42. doi: 10.1007/s00415-022-11131-w 35435464

[129] Debnath S Sharma D Chaudhari SY Sharma R Shaikh AA BuChade RS. Wheat ergot fungus-derived and modified drug for inhibition of intracranial aneurysm rupture due to dysfunction of TLR-4 receptor in Alzheimer’s disease. PloS One (2023) 18(1):e0279616. doi: 10.1371/journal.pone.0279616 36656815 PMC9851541

[130] Yamada H Umemoto T Kakei M Momomura SI Kawakami M Ishikawa SE. Eicosapentaenoic acid shows anti-inflammatory effect via GPR120 in 3T3-L1 adipocytes and attenuates adipose tissue inflammation in diet-induced obese mice. Nutr Metab (Lond). (2017) 14:33. doi: 10.1186/s12986-017-0188-0 28503189 PMC5422876

[131] Wang JH Eguchi K Matsumoto S Fujiu K Komuro I Nagai R. The ω-3 polyunsaturated fatty acid, eicosapentaenoic acid, attenuates abdominal aortic aneurysm development via suppression of tissue remodeling. PloS One (2014) 9(5):e96286. doi: 10.1371/journal.pone.0096286 24798452 PMC4010435

[132] Xia T Zhang M Lei W Yang R Fu S Fan Z. Advances in the role of STAT3 in macrophage polarization. Front Immunol (2023) 14:1160719. doi: 10.3389/fimmu.2023.1160719 37081874 PMC10110879

[133] Myers SA Gottschalk RA. Mechanisms encoding STAT functional diversity for context-specific inflammatory responses. Curr Opin Immunol (2022) 74:150–5. doi: 10.1016/j.coi.2022.01.001 35063833

[134] Zagórska A Través PG Lew ED Dransfield I Lemke G. Diversification of TAM receptor tyrosine kinase function. Nat Immunol (2014) 15(10):920–8. doi: 10.1038/ni.2986 PMC4169336 25194421

[135] Yao H Li J Liu Z Ouyang C Qiu Y Zheng X. Ablation of endothelial Atg7 inhibits ischemia-induced angiogenesis by upregulating Stat1 that suppresses Hif1a expression. Autophagy (2023) 19(5):1491–511. doi: 10.1080/15548627.2022.2139920 PMC10240988 36300763

[136] Tannahill GM Curtis AM Adamik J Palsson-McDermott EM McGettrick AF Goel G. Succinate is an inflammatory signal that induces IL-1β through HIF-1α. Nature (2013) 496(7444):238–42. doi: 10.1038/nature11986 PMC4031686 23535595

[137] Parra-Izquierdo I Castaños-Mollor I López J Gómez C San Román JA Sánchez Crespo M. Lipopolysaccharide and interferon-γ team up to activate HIF-1α via STAT1 in normoxia and exhibit sex differences in human aortic valve interstitial cells. Biochim Biophys Acta Mol Basis Dis (2019) 1865(9):2168–79. doi: 10.1016/j.bbadis.2019.04.014 31034990

[138] Elhefnawy EA Zaki HF El Maraghy NN Ahmed KA Abd El-Haleim EA. Genistein and/or sulfasalazine ameliorate acetic acid-induced ulcerative colitis in rats via modulating INF-γ/JAK1/STAT1/IRF-1, TLR-4/NF-κB/IL-6, and JAK2/STAT3/COX-2 crosstalk. Biochem Pharmacol (2023) 214:115673. doi: 10.1016/j.bcp.2023.115673 37414101

[139] Duan Z Li Z Wang Z Chen C Luo Y. Chimeric antigen receptor macrophages activated through TLR4 or IFN-γ receptors suppress breast cancer growth by targeting VEGFR2. Cancer Immunol Immunother. (2023) 72(10):3243–57. doi: 10.1007/s00262-023-03490-8 PMC10992605 37438548

[140] Meital LT Windsor MT Maynard AE Schulze K Magee R O’Donnell J. Endotoxin tolerance in abdominal aortic aneurysm macrophages, *in vitro*: A case-control study. Antioxidants (Basel) (2020) 9(9):896. doi: 10.3390/antiox9090896 32967278 PMC7554856

[141] Nishimura M. Toll-like receptor 4 expression during cerebral aneurysm formation. J Neurosurg (2013) 119(3):825–7. doi: 10.3171/2013.6.JNS09329a 23829821

[142] Zhang X Wan Y Feng J Li M Jiang Z. Involvement of TLR2/4−MyD88−NF−κB signaling pathway in the pathogenesis of intracranial aneurysm. Mol Med Rep (2021) 23(4):230. doi: 10.3892/mmr.2021.11869 33655339

[143] Li L Jin JH Liu HY Ma XF Wang DD Song YL. Notch1 signaling contributes to TLR4-triggered NF-κB activation in macrophages. Pathol Res Pract (2022) 234:153894. doi: 10.1016/j.prp.2022.153894 35489123

[144] Mandrycky CJ Abel AN Levy S Marsh LM Chassagne F Chivukula VK. Endothelial responses to curvature-induced flow patterns in engineered cerebral aneurysms. J Biomech Eng (2023) 145(1):011001. doi: 10.1115/1.4054981 35838329 PMC9445320

[145] Li M Dong X Chen S Wang W Yang C Li B. Genetic polymorphisms and transcription profiles associated with intracranial aneurysm: a key role for NOTCH3. Aging (Albany NY). (2019) 11(14):5173–91. doi: 10.18632/aging.102111 PMC6682524 31339861

[146] Tefft JB Bays JL Lammers A Kim S Eyckmans J Chen CS. Notch1 and Notch3 coordinate for pericyte-induced stabilization of vasculature. Am J Physiol Cell Physiol (2022) 322(2):C185–c96. doi: 10.1152/ajpcell.00320.2021 PMC8791789 34878922

